# Burnout among nurses: a multicentric comparative study

**DOI:** 10.1590/1518-8345.4320.3432

**Published:** 2021-06-28

**Authors:** Elisabete Maria das Neves Borges, Cristina Maria Leite Queirós, Margarida da Silva Neves de Abreu, Maria Pilar Mosteiro-Diaz, Maria Baldonedo-Mosteiro, Patrícia Campos Pavan Baptista, Vanda Elisa Andres Felli, Miriam Cristina dos Santos Almeida, Silmar Maria Silva

**Affiliations:** 1 Escola Superior de Enfermagem do Porto, CINTESIS, Porto, Portugal.; 2 Universidade do Porto, Faculdade de Psicologia e de Ciências da Educação, Porto, Portugal.; 3 Universidade de Oviedo, Departamento de Medicina, Área Enfermagem, Oviedo, Spain.; 4 Instituto de Enseñansa Secundária número 5, Departamento de Administração de Empresas, Avilés, Astúrias, Spain.; 5 Universidade de São Paulo, Escola de Enfermagem, Departamento de Orientação Profissional, São Paulo, SP, Brazil.; 6 Universidade Federal do Tocantins, Palmas, TO, Brazil.; 7 Universidade Federal de Minas Gerais, Departamento de Enfermagem Básica, Belo Horizonte, MG, Brazil.

**Keywords:** Brazil, Professional Burnout, Multicenter Study, Nursing, Portugal, Spain, Brasil, Esgotamento Profissional, Estudo Multicêntrico, Enfermagem, Portugal, Espanha, Brasil, Agotamiento Profesional, Estudio Multicéntrico, Enfermería, Portugal, España

## Abstract

**Objetivo:**

to identify and compare burnout levels between Portuguese, Spanish and Brazilian nurses.

**Method:**

quantitative, descriptive, correlational, comparative and cross-sectional study conducted using a sample of 1,052 nurses working in hospitals and primary care centers. A sociodemographic questionnaire and the Maslach Burnout Inventory were applied to nurses in Porto, Portugal (n=306), Oviedo, Spain (n=269) and S. Paulo, Brazil (n=477). Data analysis was performed using descriptive, inferential and multivariate analysis.

**Results:**

approximately 42% of the nurses showed moderate/high levels of burnout, with no differences found between countries (Portugal and Brazil 42%, Spain 43%). Only depersonalization showed differences between countries, presenting Spain the highest level and Portugal the lowest one. Comparative analysis showed higher burnout levels in young nurses and those working by shifts. Considering job schedules, burnout was associated to shift work in Portugal, while in Spain and Brazil it was associated with fixed schedules.

**Conclusion:**

these results suggest that this syndrome among nurses is a global phenomenon. The daily stressors and higher demands of the nursing profession are crucial in the preparation of nurses to deal with complex situations, to avoid burnout, and to reduce the negative impact on nurses’ health and on the quality of care they provide.

## Abstract (pt)

**Objetivo:**

identificar e comparar os níveis de *burnout* entre enfermeiros portugueses, espanhóis e brasileiros.

**Método:**

estudo quantitativo, descritivo, correlacional, comparativo e transversal, realizado com 1.052 enfermeiros em hospitais e unidades básicas de saúde. Um questionário sociodemográfico e o *Maslach Burnout Inventory* foram aplicados com enfermeiros de Porto-Portugal (n=306), Oviedo-Espanha (n=269) e São Paulo-Brasil (n=477). Os dados foram analisados por meio de estatística descritiva, inferencial e multivariada.

**Resultados:**

aproximadamente 42% dos enfermeiros apresentaram níveis moderados/altos de *burnout*, não sendo encontradas diferenças entre os países (Portugal e Brasil com 42%, Espanha com 43%). Apenas a dimensão despersonalização apresentou diferenças entre os países, com um nível mais elevado na Espanha e mais baixo em Portugal. A análise comparativa mostrou níveis mais elevados de *burnout* em enfermeiros jovens e naqueles que trabalhavam em turnos. Em relação às escalas de trabalho, *burnout* foi associada ao trabalho por turnos em Portugal e aos horários fixos na Espanha e no Brasil.

**Conclusão:**

esses resultados sugerem que essa síndrome em enfermeiros é um fenômeno global. Estressores diários e maiores demandas da profissão de enfermagem são elementos cruciais para preparar os enfermeiros para lidar com situações complexas, evitar *burnout* e reduzir o impacto negativo na sua saúde e na qualidade dos cuidados que prestam.

## Abstract (es)

**Objetivo:**

identificar y comparar los niveles de *burnout* entre enfermeros portugueses, españoles y brasileños.

**Método:**

estudio cuantitativo, descriptivo, correlacional, comparativo y transversal, realizado con 1.052 enfermeros de hospitales y unidades básicas de salud. Se aplicó un cuestionario sociodemográfico y el *Maslach Burnout Inventory* a enfermeras de Porto, Portugal (n=306), Oviedo, España (n=269) y São Paulo, Brasil (n=477). Los datos se analizaron mediante estadística descriptiva, inferencial y multivariada.

**Resultados:**

aproximadamente el 42% de los enfermeros presentaban niveles moderados/altos de *burnout* y no se encontraron diferencias entre países (Portugal y Brasil 42%, España 43%). Solo la dimensión de despersonalización mostró diferencias entre países, con un nivel mayor en España y menor en Portugal. El análisis comparativo mostró mayores niveles de *burnout* en enfermeros jóvenes y en los que trabajaban por turnos. En cuanto a los horarios de trabajo, el *burnout* se asoció con el trabajo por turnos en Portugal y con horario fijo en España y Brasil.

**Conclusión:**

estos resultados sugieren que el síndrome de *burnout* en enfermeros es un fenómeno global. Los estresores cotidianos y las mayores exigencias de la profesión de Enfermería son elementos cruciales para preparar a los enfermeros para enfrentar situaciones complejas, evitar el *burnout* y reducir el impacto negativo en la salud de los enfermeros y la calidad de la atención que brindan.

## Introduction

Since the 1970s, mostly from the studies developed in 1974 and 1976^(^ 1 ^-^ 2 ^)^, burnout syndrome is recognized as a serious professional hazard. Over the last decade, this syndrome has become more prevalent^(^ 3 ^-^ 5 ^)^ and in May 2019 was recognized it as an occupational phenomenon^(^ 6 ^)^. Thus, it is now considered a public health problem due its related consequences and has triggered considerable interest and concern in the scientific community and organizations^(^ 3 ^,^ 7 ^-^ 10). Nurses’ burnout can negatively affect the quality of care provided to patients^(^ 11 ^-^ 12 ^)^. In line with recommendations in 2018^(^ 13 ^)^ report stresses that mental health at the workplace and healthy workplaces are an increasing concern and burnout syndrome, in particular, seems to gain epidemic proportions^(^ 14 ^)^.

This syndrome is a psychological disorder triggered by chronic exposure to work stress. In 1981^(^ 15 ^)^ it was presented a consensual definition of this condition by identifying it as a three-dimensional syndrome in which the worker shows signs of emotional exhaustion (feeling powerless to provide more support to others), depersonalization (cynical and unsympathetic attitude towards patients) and low personal fulfilment (feeling of personal and professional inadequacy). Authors^(^ 16 ^-^ 17 ^)^ demonstrated that it occurs among professionals working with other persons, especially as care providers and over the years these professionals are more likely to be affected by persons’ demands.

According to the literature, health professionals are the most affected by the burnout syndrome with a higher prevalence in nurses^(^ 3 ^,^ 8 ^,^ 10 ^,^ 18). Multiple factors contribute to this phenomenon, regardless of workplace environments, and may include sociodemographic, occupational, personal characteristics, as well as established interrelationship. Numerous studies have been developed on nurses’ burnout, especially over the last years. Meta-analysis and systematic reviews^(^ 19 ^-^ 20 ^)^ highlighted the influence of job tasks, age, gender, marital status, personality traits, among others. In addition, they identified higher risk factors for professionals working in emergency or paediatric services^(^ 21 ^-^ 23 ^)^ and in primary care^(^ 24 ^)^ and nurses’ empathy-related features^(^ 25 ^)^. In addition, some studies associate burnout with turnover, ageing among nurses, nursing as a stressful occupation^(^ 26 ^-^ 30 ^)^ and, even, suicide risk among nurses^(^ 31 ^)^.

Research on burnout has been carried out in different countries. In Portugal, predictors were identified in nurses working in hospitals^(^ 10 ^)^, while authors^(^ 32 ^)^ analysed burnout among health professionals at national level and assessed its prevalence in different professional groups in hospital environments, as well as the relation between professional category and burnout levels^(^ 8 ^)^. In Spain, in a hospital, analysed the prevalence and typology of burnout syndrome in nursing professionals^(^ 33 ^)^, while other study^(^ 3 ^)^ conducted a systematic review to identify the prevalence of Emotional Exhaustion, Depersonalization and Personal Achievement of primary health care nurses. Also authors^(^ 34 ^)^ investigated burnout and stress causes among professionals working in intensive care units (physicians, nurses and assistants, showing respectively 28%, 49% and 22%). In Brazil, different studies have been developed particularly assessing the professional performance and factors associated with burnout among health professionals^(^ 5 ^)^, the prevalence of burnout predictors in nurses of an intensive care unit^(^ 35 ^)^, the relationship between burnout and depressive symptoms in nurses of an intensive care unit^(^ 36 ^)^, the association between burnout and job shifts of the nursing staff of a hospital^(^ 37 ^)^, and burnout and working environments among nurses working in public health institutions^(^ 38 ^)^.

Over the past decades there has been an increase of cross-cultural methods in research addressing workplaces and organizations, since it allows understanding and dealing with both differences and common patterns in different cultural contexts^(^ 7 ^,^ 39 ^-^ 40). Due to the historical background, geographical, cultural or language proximity, Portugal, Brazil and Spain share many characteristics that facilitate the regular movement of professionals between these countries. Therefore, it is important to develop comparative studies and an European report published by the Agency for the Improvement of Living and Working Conditions^(^ 41 ^)^ emphasized that despite “burnout has been the subject of research and policy responses across Europe”, it is important that we gain “an EU (European Union) wide perspective on the issue”.

This study aims to identify and compare burnout levels of Portuguese, Spanish and Brazilian nurses.

## Method

A quantitative, descriptive, correlational, comparative and cross-sectional study was conducted using a sample of 1,052 nurses, being 306 from Porto, Portugal, 269 from Oviedo, Spain and 477 from S. Paulo, Brazil, through an intentional sampling and the snowball technique. Participants were all working in public hospitals and public health centres and the inclusion criteria were: to be actively employed and with a job experience of more than 6 months. Data collection was conducted between 2016 and 2017. Considering the whole sample, 83% were female, with an average age of 37 years, 58% had a marital partner, 60% worked in hospital settings, 56% worked fixed shifts and 58% had less than 13 years of job experience (Table 1).

**Table 1:** Social demographic characteristics of nurses (1,052). Portugal, Spain and Brazil, 2016-2017

		Total		Portugal		Spain		Brazil		P*
		N	%	n	%	N	%	n	%	
	Total	1052	100	306	29.1	269	25.6	477	45.3	
Gender	Male	177	16.8	92	30.1	41	15.2	44	9.2	<0.001
	Female	875	83.2	214	69.9	228	84.8	433	90.8	
Average age (SD)^†^		37.4 (9.1)		34.6 (8.6)		40.8 (9.2)		37.4 (8.7)		<0.001
Marital status	Without marital partner	443	42.1	159	52.0	106	39.4	178	37.3	<0.001
	With marital partner	609	57.9	147	48.0	163	60.6	299	62.7	
Workplace	Hospital	634	60.3	196	64.1	190	70.6	248	52.0	<0.001
	Health Centre	291	27.7	77	25.2	0	0.0	214	44.9	
	Other	127	12.1	33	10.8	79	29.4	15	3.1	
Professional category	Nurse	706	67.1	306	100.0	269	100.0	131	27.5	<0.001
	Nurse assistant	346	32.9	0	0.0	0	0.0	346	72.5	
Shift	Fixed	585	55.6	108	35.3	13	4.8	464	97.3	<0.001
	Rotating	467	44.4	198	64.7	256	95.2	13	2.7	
Professional experience	<13 years	614	58.4	196	64.1	101	37.5	317	66.5	<0.001
	≥13 years	438	41.6	110	35.9	168	62.5	160	33.5	

* value obtained through the Chi-square test and ANOVA;

† SD = Standard deviation

A sociodemographic and professional questionnaire (gender, age, marital status, country, workplace, professional category, shift work and professional experience), was used to collect data. To assess burnout it was used the Maslach Burnout Inventory-Human Services Survey (MBI-HSS) translated and adapted for the Portuguese, Spanish and Brazilian population^(^ 15 ^,^ 42 ^-^ 45). This instrument includes 22 items, recorded on a Likert scale ranging from 0 (never) to 6 (every day) and organized in three dimensions: Emotional Exhaustion (9 items), Depersonalization (5 items) and Personal Achievement (8 items). The total score of the instrument was used to calculate burnout levels considering the inverted items of Personal Achievement and all scores were calculated considering the average of all related items. It was also possible to classify individuals according to the level of burnout, based on the following cut-off points^(^ 32 ^)^: <2, no burnout; [2,3[, moderate burnout; ≥3, high burnout.

No direct contact was established between researchers and participants and the questionnaires were sealed and delivered by one of the researchers at the previously set locations and date and after anonymously completed by the participants (whenever possible the questionnaires were filled in at the time of delivery to the workplace, considering the participant’s availability, and the period of a month was extended for an equal period to complete the questionnaires) they were collected for analysis. To allow comparative studies, the same procedures were adopted in all countries involved in this study and standard surveys, namely no individual results disclosure to the institutions.

The study was approved by the Ethics Committees of Nursing School of Porto (8/2016), and University Hospital of the University of São Paulo, Brazil and Regional Clinical Research Ethics Committee of the Principality of Asturias, Oviedo, Spain. Formal consents were granted by the institutions involving nurses’ participation and all participants were asked to sign an informed consent.

Data analysis was performed using the SPSS 24 software with a significance level of 0.05 in all analyses. Descriptive analysis of the data was performed, considering absolute and relative frequencies, median or median and standard deviation and interquartile range.

Normality was tested through the Kolmogorov-Smirnov test. The Chi-square of Pearson test and Analysis of variance ANOVA were applied to compare the participants’ characteristics according to country. Kruskall-Wallis test was used when the assumption of normality was not verified. The Scheffe test or the Dunn’s test were used for multiple comparisons of the ANOVA or Kruskall-Wallis, respectively.

To identify potential predictors of the quantitative dependent variables under analysis and with normal distribution, mixed linear models were used, considering the country as a random effect (based on the multi-level nature of the study). In a first step, univariate models were used (considering one factor at a time), in order to identify potential predictive factors of each dependent variable. Based on these results, a multivariate model for each dependent variable was developed, with all independent related variables obtained from the univariable models (except for “workplace” and “professional category”, because data were not available for the three countries). Finally, the country’s interactions with the independent variables were tested. Only the significant results were presented.

## Results

To perform comparative analyses with models adjusted for the burnout results, the total and country sociodemographic and professional variables were identified. Statistically significant differences were found between countries for all studied variables (Table 1). Portugal showed the highest percentage of male participants, with no marital partner; the average age of participants was lower in Portugal and higher in Spain. No nurses were working in health centres in Spain. In Brazil, nursing assistants were included in the sample, since nurses perform tasks similar to Portugal and Spain. Almost all participants work rotating shifts in Spain and in Brazil, the most common is the fixed shift. Spanish participants had more professional experience. The Scheffe test revealed significant differences between all countries for mean age.

Concerning burnout, Table 2 presents the median scores and standard deviations for the total burnout scale and its dimensions, for the total sample and *per* country, as well as the distribution of participants according to the categories of the burnout level, for the total sample and per country. A large percentage of nurses showed moderate/high burnout levels (42%, 43% and 42%, respectively in Portugal, Spain and Brazil) and higher values of Emotional Exhaustion and Personal Achievement than of Depersonalization. Only the Depersonalization dimension showed differences between countries (p<0.001). No statistically differences were found between countries on the remaining dimensions and total score (p>0.05). Spain scored the highest for the Depersonalization dimension [median=1.60 (IQI-Interquartile interval=1.80)] and Portugal received the lowest score [median=0.60 (IQI=1.20)]. The multiple comparisons test revealed that all countries were distinct from each other (p<0.05 when comparing all pairs).

**Table 2:** Comparative analysis of burnout and dimensions in nurses (1,052). Portugal, Spain and Brazil, 2016-2017

Dimensions	All sample		Portugal		Spain		Brazil		p	p^§^	p^||^	p^¶^
	Mean (SD)^†^		Mean (SD)^†^		Mean (SD)^†^		Mean (SD)^†^					
Emotional exhaustion	2.54 (1.35)		2.68 (1.30)		2.46 (1.22)		2.50 (1.44)		0.093	-	-	-
Depersonalization*	1.00 (1.80)		0.60 (1.20)		1.60 (1.80)		1.00 (1.80)		<0.001^‡^	0.033	<0.001	<0.001
Personal achievement	4.52 (1.01)		4.53 (0.86)		4.54 (0.98)		4.50 (1.12)		0.851	-	-	-
Burnout	1.87 (0.89)		1.86 (0.83)		1.90 (0.87)		1.85 (0.93)		0.759	-	-	-
	n	%	n	%	n	%	n	%				
Burnout levels												
Absence	608	57.8	177	57.8	153	56.9	278	58.3	0.784			
Moderate	326	31.0	100	32.7	84	31.2	142	29.8				
High	118	11.2	29	9.5	32	11.9	57	11.9				

* Median (IQI=Interquartile interval);

† SD = Standard deviation;

‡ Value obtained through the Kruskal-Wallis test;

§ Pairwise comparisons between Portugal and Brazil;

|| Pairwise comparisons between Portugal and Spain;

¶ Pairwise comparisons between Spain and Brazil

Table 3 shows the coefficients and standard errors estimated for the independent variables under analysis, considering the univariate models and the multivariate model (adjusted model). From the data analysis, it is possible to verify that the variables age and shift are significant predictors of burnout, remaining significant in the adjusted model. Older participants with a fixed shift report lower burnout levels compared to younger participants with a rotating shift, controlling for the remaining variables.

**Table 3:** Univariate models and adjusted model of the nurses' burnout (1,052). Portugal, Spain and Brazil, 2016-2017

Variable	Categories	Not-adjusted		Adjusted	
		Coefficient (ep)	p	Coefficient (ep)	p
Country	Portugal	0.012 (0.89)	0.990	-0.153 (0.891)	0.864
	Spain	0.050 (0.89)	0.955	-0.115 (0.893)	0.897
	Brazil	0	-	0	-
Gender	Male	0.073 (0.07)	0.320	0.078 (0.075)	0.304
	Female	0	-	0	-
Age		-0.008 (0.003)	0.011	-0.010 (0.004)	0.024
Marital status	Without marital partner	0.062 (0.055)	0.264	0.026 (0.057)	0.650
	With marital partner	0	-	0	-
Place	Hospital	0.170 (0.086)	0.049		
	Health Centre	0.009 (0.094)	0.923		
	Other	0	-		
Professional category	Nurse	0.051 (0.058)	0.386		
	Nurse assistant	0	-		
Shift	Fixed	-0.122 (0.057)	0.033	-0.189 (0.093)	0.043
	Rotating	0	-	0	-
Professional experience	<13 years	0.058 (0.056)	0.296	-0.064 (0.076)	0.401
	≥13 years	0	-	0	-
	Fixed Portugal			-0.536 (0.27)	0.048
	Fixed Spain			-0.046 (0.35)	0.897
	Random effect	0.790 (0.034)	<0.001	0.784 (0.034)	<0.001

**Figure 1:**
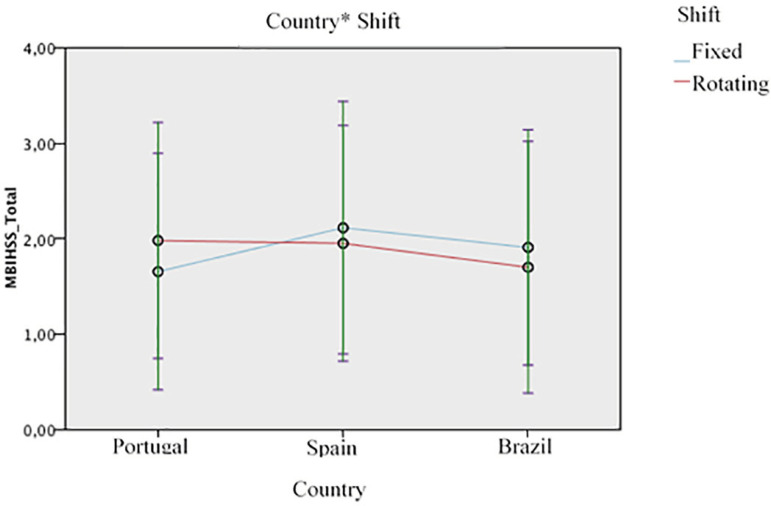
Interaction effect of country-shift work on nurses’ burnout (1,052). Portugal, Spain and Brazil, 2016-2017 (based on the multivariable model)

Considering the multivariable model presented at Table 3 and including a covariate composed by the interaction between country and shift variable, a statistically significant interaction between these variables was found (Figure 1), showing the shift effect on burnout to be different depending on the country. In Portugal, the rotating shift is associated with a higher level of burnout, contrary to Brazil and Spain, where the fixed shift is associated with a higher level of burnout. Results of the other main effects remain similar.

Regarding Emotional Exhaustion (Table 4), the coefficients and standard errors estimated for the independent variables under analysis, were calculated, considering the univariate models. The analysis showed that no tested variable was a predictive factor of emotional exhaustion, and, considering this result, it was decided not to develop the adjusted model.

**Table 4:** Emotional exhaustion, depersonalization and personal achievement: univariate models and adjusted model in nurses (1,052). Portugal, Spain and Brazil, 2016-2017

Variable	Categories	Emotional exhaustion		Depersonalization				Personal achievement				
		Not-adjusted		Not-adjusted		Adjusted		Not-adjusted			Adjusted	
		Coefficient (se)*	p	Coefficient (se)*	P	Coefficient (se)*	p	Coefficient (se)*	p	Coefficient (se)*		p
Country	Portugal	0.180 (1.348)	0.894	-0.225 (1.14)	0.844	-0.503 (1.14)	0.658	0.028 (1.017)	0.978	0.083 (1.017)		0.935
	Spain	-0.042 (1.348)	0.975	0.368 (1.14)	0.747	0.061 (1.14)	0.957	0.042 (1.018)	0.967	-0.007 (1.020)		0.995
	Brazil	0	-	0	-	0	-	0	-	0		-
Gender	Male	0.005 (0.113)	0.068	0.201 (0.10)	0.037	0.202 (0.096)	0.036	-0.087 (0.084)	0.298	-0.115 (0.086)		0.180
	Female	0	-	0	-	0	-	0	-	0		-
Age		-0.007 (0.005)	0.148	-0.008 (0.004)	0.035	-0.008 (0.005)	0.129	0.011 (0.003)	0.001	0.011 (0.005)		0.023
Marital status	Single	0.017 (0.084)	0.839	0.089 (0.072)	0.212	0.050 (0.073)	0.496	-0.107 (0.063)	0.090	-0.075 (0.065)		0.249
	With marital partner	0	-	0	-	0	-	0	-	0		-
Place	Hospital	0.161 (0.133)	0.227	0.319 (0.11)	0.005			-0.153 (0.098)	0.120			
	Health Centre	0.092 (0.152)	0.548	0.137 (0.13)	0.309			0.040 (0.108)	0.714			
	Other	0	-	0	-			0	-			
Professional category	Nurse	0.102 (0.112)	0.361	0.268 (0.11)	0.019			0.070 (0.067)	0.291			
	Nurse assistant	0	-	0	-			0	-			
Shift	Fixed	-0.229 (0.120)	0.057	-0.380 (0.11)	0.001	-0.332 (0.119)	0.005	-0.001 (0.063)	0.992	-0.015 (0.107)		0.889
	Rotating	0	-	0	-	0	-	0	-	0		-
Professional experience	<13 years	0.009 (0.086)	0.918	0.077 (0.073)	0.296	-0.054 (0.097)	0.577	-0.154 (0.063)	0.015	-0.017 (0.087)		0.846
	≥13 years	0	-	0	-	0	-	0	-	0		-
	Random effect	1.811 (0.079)	<0.001	1.296 (0.057)	<0.001	1.279 (0.056)	<0.001	1.030 (0.045)	<0.001	1.022 (0.045)		<0.001

* (se) = Standard error

Despite the asymmetry found in the variable Depersonalization (through the analysis of the histogram by country), the index of asymmetry varied between 0.213 and 1.533 and the index of flattening varied between -0.218 and 2.367. According to these indices, the variable was considered symmetric and the model described above was used. In Table 4 it is possible to observe the variables gender, age workplace, professional category and shift as significant predictors of Depersonalization, with variables gender and shift remaining significant in the adjusted model. Male participants show higher Depersonalization levels compared to female participants, controlling for the remaining variables. In addition, participants with a fixed shift report lower Depersonalization levels compared to those with a rotating shift, controlling for the remaining variables. After testing the interactions between the country and the variables gender and shift, no statistically significant interaction were found between them.

Regarding Personal Achievement (Table 4), the coefficients and standard errors estimated for the independent variables under analysis were calculated, considering the univariate models and multivariate model (adjusted model), showing age and professional experience as significant predictors of Personal Achievement. However, only the variable age remains significant in the adjusted model and older participants show a higher level of Personal Achievement, controlling the remaining variables. The interaction between country and age is significant, since for Portuguese and Spanish participants, as they get older, lower scores in Personal Achievement are obtained, compared to Brazil.

## Discussion

These study findings revealed a large percentage of nurses with moderate/high burnout levels (42%, 43% and 42%, respectively in Portugal, Spain and Brazil) and higher scores on Emotional Exhaustion and Personal Achievement than for Depersonalization. One study^(^ 36 ^)^ revealed that only 14% of Brazilian nurses reported burnout levels. However, the cross-cultural study among Portuguese and Brazilian nurses found that in the Emotional Exhaustion and Personal Achievement dimensions, nurses from both countries showed moderate and high values, respectively^(^ 7 ^)^. These findings are explained by the differences in the type and work contexts. It should be noticed that in Portugal nurses spend most of their time providing direct care to patients and it is expected that they establish a strong relationship with the patient and demonstrate high technical competence. Moreover, in Portugal, a study^(^ 8 ^)^ found that the Emotional Exhaustion dimension scored the highest values in the majority of nurses (59%). Regarding Spain, in the systematic review with primary care nurses, the authors found that 50% of nurses had low/medium levels on Emotional Exhaustion and 50% had high levels^(^ 3 ^)^.

It was also found that older nurses in all countries and those working in shifts in Spain and Brazil showed lower burnout levels. Another study^(^ 35 ^)^ corroborate these results, as they found that the burnout syndrome was higher for individuals aged between 22 and 29 years. These authors report that young professionals are considered inexperienced and more likely to feel anxiety when dealing with complex and unknown situations. Contrarily, in Spain, highlighted that longer professional experience may be related to the age of professionals and found that professionals with medium and high professional experience showed the highest percentage of burnout^(^ 33 ^)^. In addition, the syndrome occurs in two periods: in the first two years of the professional career and following 10 years of experience^(^ 33 ^)^. Moreover, in Spain, found that older nurses with longer working experience had higher levels of burnout^(^ 3 ^)^. In the study addressing shift work in Brazil, found equal percentages of burnout in professionals with fixed and rotating work^(^ 36 ^)^.

Regarding the dimensions Emotional Exhaustion, Depersonalization and Personal Achievement, in this study, it was possible to observe that gender, age, workplace, professional category and shift predicted Depersonalization, while age and professional experience predicted Personal Achievement. There was an association between Depersonalization and gender, with Depersonalization scoring higher for women. Depersonalization in nurses also showed a significant association with schooling. In addition, higher levels of professional achievement were associated with professionals with post-graduate training^(^ 5 ^)^. That Emotional Exhaustion was related to the institutions with more unfavourable working conditions regarding autonomy, organisational support and environmental control^(^ 38 ^)^.

Study findings showed that Emotional Exhaustion and Low Professional Achievement levels were significantly higher among nurses with daytime shifts^(^ 37 ^)^. This could be explained by the workload of nursing care and procedures during this period. Also, the interpersonal relationship with the multiprofessional team is often more frequent, increasing occupational stress and the development of the burnout. The importance of the workplace in the development of burnout syndrome alerted for the differences between the working day of an emergency or intensive care nurse with that of primary health care nurses^(^ 3 ^)^. In primary health care services, nurses work in prevention, education, follow-up and prolonged and continuous treatment of the population, mainly focused on chronic pathologies. Special attention is also given to the community and home-dwelling more prolonged interventions, compared to the short duration of acute diseases common to hospital services. This highly explains different levels of burnout across services.

A study^(^ 46 ^)^ found that women reported higher levels of burnout, although with no significant differences, while men experienced higher levels of Depersonalization, with Emotional Exhaustion predicted by professional experience and gender (higher in the most experienced participants and women). Regarding Depersonalization, the variance was only explained by gender and women were less likely to evidence symptoms of Depersonalization than men. These results can be due to the way that man and women deal with their own emotions and job emotional demands.

There are several examples of comparative studies, such as the cross-cultural study about the influence of hardiness in burnout syndrome among Brazilian and Portuguese nurses^(^ 7 ^)^. Some authors^(^ 39 ^)^ studied the association between fast food and alcohol consumption, physical exercise and analgesic use in a multinational sample of 2,623 physicians and nurses living in Greece, Portugal, Bulgaria, Romania, Turkey, Croatia and Macedonia. Another study including health professionals from Spain and Spanish-speaking Latin American countries, identified the frequency and intensity of the perception of the adverse consequences of the profession and its association with burnout syndrome and professional variables^(^ 40 ^)^.

Comparative studies are still difficult to perform, despite the fact that we currently live in a globalization world where nurses are constantly facing the same challenge and threats worldwide^(^ 47 ^)^. In fact, these professionals need to work and decide under stressful and pressure environments, while interacting with patients and their families in situations often characterized by strong emotional distress. Additionally, nurses experience changes in the family relationship^(^ 48 ^)^, leading to less job satisfaction and more turnover and disengagement^(^ 3 ^)^.

The World Health Organization^(^ 6 ^)^ has recently acknowledged burnout syndrome as a disease-related employment problem. In addition, the COVID-19 pandemic triggered new challenges, with profound impact on nurses well-being, namely increased stress levels, post-traumatic stress and burnout^(^ 49 ^-^ 50 ^)^.

Despite being a multicentre study, the generalisability of these results is not recommended, mainly because it is a cross-sectional study and based on voluntary participation though the application of a self-administered questionnaire with data collection being performed in specific regions of each country. Although these countries share many similarities, the differences found stress the importance of conducting longitudinal and randomized studies involving other nurses’ working environments. It is also suggested that the dimension emotional exhaustion is further investigated to test other variables that may explain this dimension.

As a result of research on burnout and due to the high percentage of Portuguese, Spanish and Brazilian nurses with moderate/high burnout levels and the significant financial burden for health institutions, nurses, family and society, it is imperative that policy makers continue to invest in the area of occupational health. Importantly, health services administrators must develop infrastructures that promote the occupational health and well-being of nurses. We strongly believe that investing on the academic curricula with special emphasis on burnout and other work-related risks may likely lead to improved well-being, safety, quality of care and overall health of populations. Also, the development of multicentric research provides a strong contribution to scientific nursing knowledge.

## Conclusion

During the professional activity, nurses are exposed to numerous and multiple combining stressors, very likely to have a negative impact on the professional and the organization, with special highlight to burnout syndrome. Approximately 40% of nurses showing burnout levels were found in each country. Therefore, it is important to prepare nurses to identify the risks of developing burnout and help them to find resources within the family, the community and the organization in order to improve their well-being. Considering the impact of burnout, a future randomised controlled trial should be conducted to include a programme involving work contexts with potentially high levels of stress.
